# Lead-rich carboxylate-substituted titanium–lead oxo clusters

**DOI:** 10.1007/s00706-017-1972-2

**Published:** 2017-06-20

**Authors:** Christine Artner, Ulrich Schubert

**Affiliations:** Institute of Materials Chemistry, Vienna University of Technology, Getreidemarkt 9, 1060 Vienna, Austria

**Keywords:** Metal oxo clusters, Lead compounds, Titanium compounds

## Abstract

**Abstract:**

The carboxylate-substituted mixed-metal oxo clusters Pb_6_Ti_6_O_9_(acetate)(methacrylate)_17_ and Pb_4_Ti_8_O_10_(O*i*Pr)_18_(acetate)_2_ contain a higher number of lead atoms in the cluster core than previously reported compounds. The metal atoms in both clusters are arranged in three layers of different composition, which are connected through oxygen, propionate and/or carboxylate bridges.

**Graphical abstract:**

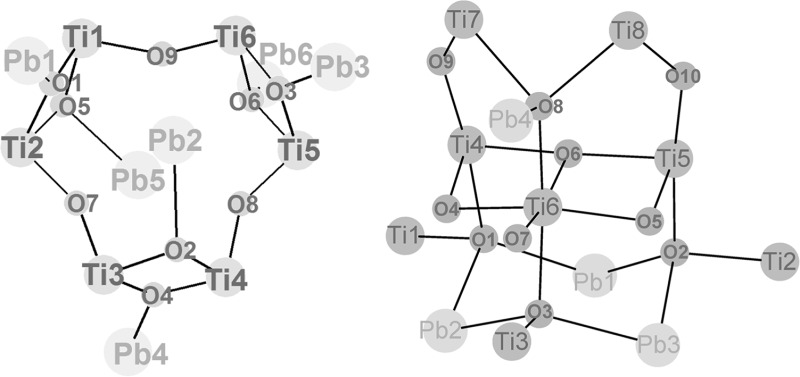

## Introduction

Metal oxo clusters of the general composition M_a_O_b_(OH/OR)_c_(OOCR′)_d_ are obtained when early transition metal alkoxides, M(OR)_n_, are reacted with more than one molar equivalent of carboxylic acid [1]. The carboxylic acid not only provides carboxylate ligands but also the oxo groups through esterification with the alcohol eliminated during the substitution reaction. This protocol can be extended to mixed-metal oxo clusters by employing mixtures of metal alkoxides or, alternatively, a metal alkoxide and a metal salt.

A variety of titanium/metal oxo clusters has been obtained by this route. The structures of some of them are based on common structural motives, as has been discussed elsewhere in detail [2, 3]. Carboxylate-substituted Pb/Ti oxo clusters are in a sense unique as a variety of compounds with different Pb:Ti proportions are known. This allows gaining insight in how the structural features depend on the Ti/metal ratio. Carboxylate-substituted mixed-metal clusters generally have a richer structural chemistry than polynuclear compounds with a similar composition but without such ligands, because the bridging ligands provide more possibilities for connecting metals. Thus, contrary to the many examples of carboxylate-substituted Pb/Ti oxo clusters, only one unsubstituted cluster is known, viz. Pb_2_Ti_2_O(O*i*Pr)_10_, the structure of which is based on a Pb_2_Ti_2_(μ_4_-O) tetrahedron [4].

The (chain-like) structures of the carboxylate-substituted oxo clusters with a Pb:Ti ratio of 2:2–4, Pb_2_Ti_2_O(O*i*Pr)_8_(OAc)_2_ (**Pb2Ti2**) [5], Pb_2_Ti_3_O_2_(O*i*Pr)_12_(OOCC_7_H_15_)_2_ (**Pb2Ti3**) [6], and Pb_2_Ti_4_O_2_(OEt)_14_(OAc)_2_ (**Pb2Ti4**) [7], are based on Ti_2_(OR)_x_(μ-OOCR)_y_ building blocks. The polynuclear compounds Pb_2_Ti_4_(OR)_16_(OOCR′)_4_ [6, 8] (without oxo groups, with different R and R′) have similar structures to that of **Pb2Ti4**.

The structures of the clusters Pb_2_Ti_8_O_8_(OBu)_2_X_2_(OMc)_16_(BuOH)_2_ (**Pb2Ti8**, X = OAc or OMc (OMc = methacrylate)) and Pb_2_Ti_6_O_5_(O*i*Pr)_3_X(OMc)_14_ (X = O*i*Pr or OMc) (**Pb2Ti6**) [9], with a higher Ti proportion, however, are derived from cyclic Ti_8_O_8_(OOCR)_16_. In the latter compound, each Ti atom is connected to both neighbouring Ti atoms through one *μ*_2_-oxygen and two bridging OMc ligands each. The Ti_8_O_8_ ring has crown ether-like properties. In **Pb2Ti8**, two Pb(II) ions occupy the central cavity. Coordination of the oxygen atoms of the Ti_8_O_8_ ring to Pb is supported by bridging carboxylate ligands. In **Pb2Ti6**, the central Pb_2_ unit is coordinated by a semi-circular Ti_6_ fragment of the Ti_8_O_8_(OMc)_16_ metallacycle.

Common to the known carboxylate-substituted Pb/Ti oxo clusters is the metal ratio of Pb_2_Ti_x_ (*x* = 2–4, 6, 8), notwithstanding the different structures, the different metal:oxygen ratios and the different ligand shell composition. In this article we report two new Pb/Ti oxo clusters with a greater number of lead atoms, viz. Pb_6_Ti_6_O_9_(OAc)(OMc)_17_ (**Pb6Ti6**) and Pb_4_Ti_8_O_10_(O*i*Pr)_18_(OAc)_2_ (**Pb4Ti8**).

## Results and discussion

Metal oxo clusters are very reproducibly obtained when all reaction parameters are meticulously kept, whereas seemingly minor variations may result in different clusters. For example, crystals of **Pb2Ti8** were formed within three weeks when Pb(OAc)_2_, Ti(OBu)_4_, and methacrylic acid were reacted in a 1:1:4 ratio at room temperature [9]. In contrast, colourless crystals of **Pb6Ti6** were obtained after four months when equimolar amounts of Pb(OAc)_2_ and Ti(O*i*Pr)_4_ were first reacted at 70 °C in allylic alcohol, and two equivalents of methacrylic acid were added after cooling. The same reaction at room temperature resulted in the same cluster as **Pb2Ti8** with OAllyl instead of OBu ligands. Note that **Pb6Ti6** contains no residual OR ligands as in the other PbTi clusters.

**Fig. 1:**
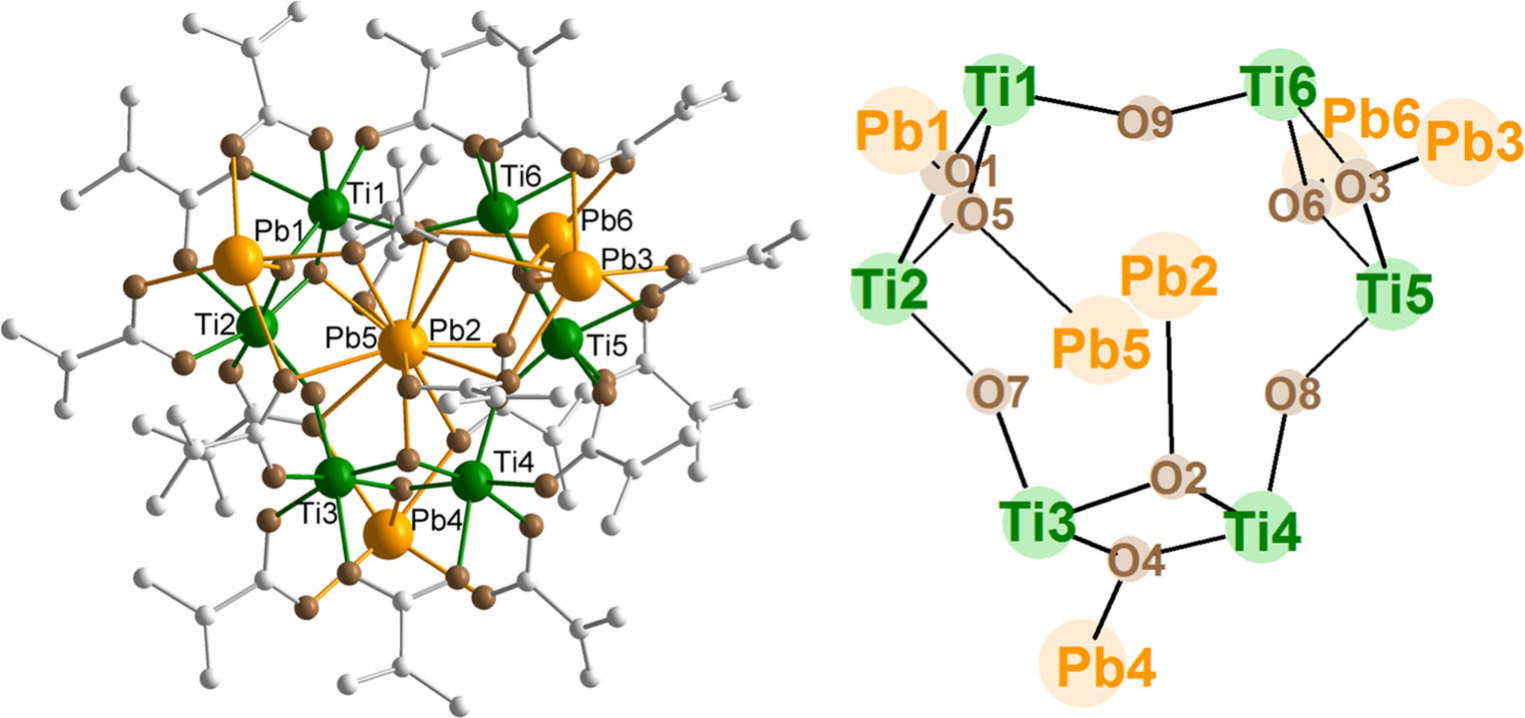
*Left* Molecular structure of Pb_6_Ti_6_O_9_(OAc)(OMc)_17_. Hydrogen atoms are omitted for clarity. *Right* Cluster core

The structure of **Pb6Ti6** (Fig. 1; Table 1) represents an interesting variation of the “Ti_8_O_8_ ring” motif, as it is based on a Ti_6_O_6_ ring as the central structural feature. While a great variety of Ti_8_O_8_(OOCR)_16_ (= [TiO(OOCR)_2_]_8_) structures is known (with different carboxylate ligands), Ti_6_O_6_ rings are not known, although a [TiO(OOCR)_2_]_6_ ring system appears to be stereochemically possible. The central unit of **Pb6Ti6** comes close to such a structure (Fig. 1, right). The six titanium atoms are alternatively bridged by one or two oxygen atoms (whereas there is only one bridging oxygen between neighbouring Ti atoms in **Pb2Ti8)**. The *μ*_2_-oxygen atoms O(7)–O(9) are located within the Ti_6_ plane.

**Table 1:** Selected bond lengths/Å and angles/° for **Pb6Ti6**

Metal–metal distances	
Ti(1)–Ti(2) 2.8522(8)	Ti(4)–Ti(5) 3.4930(10)
Ti(2)–Ti(3) 3.5914(10)	Ti(5)–Ti(6) 2.9370(9)
Ti(3)–Ti(4) 2.8398(8)	Ti(1)–Ti(6) 3.5041(9)
Pb(1)–Pb(2) 3.9100(6)	Pb(4)–Pb(5) 3.9884(9)
Pb(2)–Pb(3) 4.0036(11)	Pb(5)–Pb(6) 3.932(2)
Pb(2)–Pb(5) 4.5535(7)	
Metal-*μ*_3_-O distances	
Ti(1)–O(1) 1.901(2)	Ti(4)–O(2) 1.850(2)
Ti(1)–O(5) 1.831(2)	Ti(4)–O(4) 1.895(2)
Ti(2)–O(1) 1.932(2)	Ti(5)–O(3) 1.930(2)
Ti(2)–O(5) 1.900(2)	Ti(5)–O(6) 1.902(2)
Ti(3)–O(2) 1.873(2)	Ti(6)–O(3) 1.909(2)
Ti(3)–O(4) 1.931(2)	Ti(6)–O(6) 1.936(2)
Pb(1)–O(1) 2.284(2)	Pb(4)–O(4) 2.277(2)
Pb(2)–O(2) 2.509(2)	Pb(5)–O(5) 2.519(2)
Pb(3)–O(3) 2.244(3)	Pb(6)–O(6) 2.262(3)
Metal-*μ*_2_-O distances	
Ti(1)–O(9) 1.868(2)	Ti(4)–O(8) 1.856(2)
Ti(2)–O(7) 1.837(2)	Ti(5)–O(8) 1.810(2)
Ti(3)–O(7) 1.841(2)	Ti(6)–O(9) 1.799(2)
Metal-*μ*_3_-O-metal angles	
Ti(1)–O(1)–Ti(2) 96.15(10)	Ti(3)–O(2)–Ti(4) 99.42(11)
Ti(1)–O(5)–Ti(2) 99.70(10)	Ti(5)–O(3)–Ti(6) 99.79(11)
Ti(3)–O(4)–Ti(4) 95.85(10)	Ti(5)–O(6)–Ti(6) 99.84(11)
Pb(1)–O(1)–Ti(1) 133.22(11)	Pb(4)–O(4)–Ti(3) 126.56(11)
Pb(1)–O(1)–Ti(2) 127.53(11)	Pb(4)–O(4)–Ti(4) 133.84(11)
Pb(2)–O(2)–Ti(3) 109.81(10)	Pb(5)–O(5)–Ti(1) 124.18(11)
Pb(2)–O(2)–Ti(4) 118.20(10)	Pb(5)–O(5)–Ti(2) 106.73(9)
Pb(3)–O(3)–Ti(5) 123.39(12)	Pb(6)–O(6)–Ti(5) 128.74(15)
Pb(3)–O(3)–Ti(6) 128.60(13)	Pb(6)–O(6)–Ti(6) 121.98(14)
Ti-*μ*_2_-O-Ti angles	
Ti(2)–O(7)–Ti(3) 155.13(13)	Ti(1)–O(9)–Ti(6) 145.69(13)
Ti(4)–O(8)–Ti(5) 144.63(14)	

The six Pb atoms are arranged in two layers of three Pb atoms each above and below the Ti_6_ plane (Fig. 1, right). The three metal planes are almost parallel to each other. Each Pb atom is coordinated to one oxygen atom of the Ti_2_O_2_ units. Hence, these oxygen [O(1)–O(6)] atoms are *μ*_3_, connecting two Ti and one Pb atom. The Pb–O axes, however, have different orientations. While four [Pb(1), Pb(3), Pb(4) and Pb(6)] point away from the ring centre, the other two Pb atoms [Pb(2) and Pb(5)] are located above and below the centre of the Ti_6_ ring. Pb(3), Pb(4), and Pb(6) show positional disorder, but this does not affect the ligands. While each Pb atom is connected to two Ti atoms through a *μ*_3_-O, only the outer Pb atoms are additionally connected to the Ti layer by bridging ligands (see below). The Pb-*μ*_3_-O bonds of the central lead atoms Pb(2) and Pb(5) are significantly longer [Pb(2)–O(2) 2.509(2) Å, Pb(5)–O(5) 2.519(2) Å] than that of the outer Pb atoms [2.244(3)–2.284(2) Å].

Pb^2+^ has a lone pair of electrons, which is often stereochemically active. This is indicated by truncated coordination polyhedra of the corresponding metals. In **Pb6Ti6**, the lone pairs of the outer Pb atoms [Pb(1), Pb(3), Pb(4), and Pb(6)] point away from the cluster centre. In contrast, the lone pairs of Pb(2) and Pb(5) point to the centre the Ti_6_ ring and towards each other. The Pb(2)···Pb(5) distance of 4.5535(7) Å is relatively short for a Pb···Pb distance of non-bridged lead atoms. This placement of two lead atoms above and below the centre of the Ti_6_ ring at a short distance is apparently very favourable. The sum of bond angles around the *μ*_3_-oxygen atoms support this assumption: while O(2) and O(5) are clearly pyramidal [Σ M–O(2)–M 327.4(3)° and M–O(5)–M 330.6(3)°], indicating some pulling of the lead atoms towards the ring centre, the other *μ*_3_-oxygen atoms are planar [350.6(4)–356.9(3)].

**Fig. 2:**
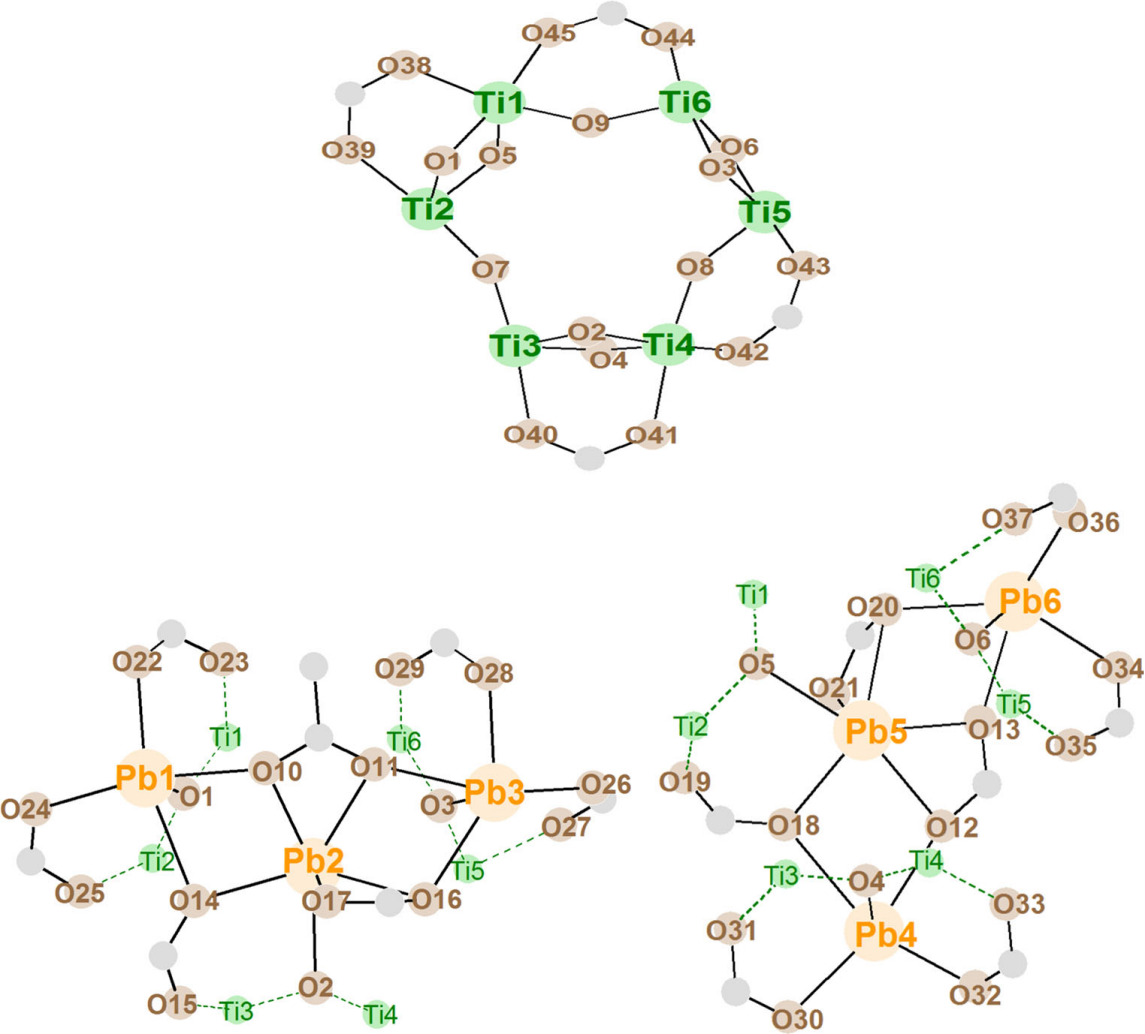
Schematic drawing of the three metal layers of **Pb6Ti6**. Only the COO groups of the OMc ligands are drawn. *Dashed lines* indicate bonds to metals of adjacent layers

The positioning of Pb(2) and Pb(5) above and below the Ti_6_ ring centre at a short Pb···Pb distance is probably the reason for the non-centrosymmetric positions of the other lead atoms and the ligand shell around the Ti_6_ ring (Fig. 2). Only Ti(1) and Ti(4) have OMc bridges to both neighbouring Ti atoms, but there is no methacrylate bridge between Ti(2) and Ti(3), or Ti(5) and Ti(6). The central Ti_6_ ring system and the attached Pb atoms are approximately C_2_-symmetric, with the axis of rotation passing through O(3) and the centre of the Ti_2_O_2_ ring, formed by O(6), O(7), Ti(5), and Ti(6). The pairs of Ti atoms which are not connected by a OMc bridge have instead OMc bridges to the adjacent Pb_3_ layers. Ti(2) and Ti(3) are connected to one Pb_3_ layer through a *μ*_3_-OMc ligand each and to the other by a *μ*_2_-OMc ligand. Thus, Ti(3) is connected to Pb(1) and Pb(2) of the top layer through a *μ*_3_-OMc ligand and to Pb(4) of the bottom layer through a *μ*_2_-OMc ligand. Conversely, Ti(2) is connected to Pb(4) and Pb(5) of the bottom layer and to Pb(1) of the top layer. On the other side of the Ti_6_ ring, Ti(5) and Ti(6) are connected to one Pb atom of both adjacent Pb_3_ layers (Pb(3) and Pb(6)) by two OMc bridges. The two Ti atoms with two OMc bridges to both neighbouring Ti atoms also connect to the Pb_3_ layers by one OMc bridge each [Ti(1)··Pb(1) and Ti(4)··Pb(4)].

The remaining four carboxylate ligands connect the Pb atoms in the Pb_3_ layers. One carboxylate group bridges all three Pb atoms. It is chelating the central Pb atom [Pb(2) or Pb(5)] and bridging to both other Pb atoms of the layer. In the top layer this carboxylate ligand is an acetate group, but a methacrylate ligand in the bottom layer. Each of the other two OMc ligands is again chelating the central Pb atom [Pb(2) or Pb(5)] and bridges only to one other Pb atom.

**Pb6Ti6** co-crystallizes with an allylic alcohol molecule which weakly interacts with Pb(6), with a Pb–O distance of 3.12(2) Å.

**Fig. 3:**
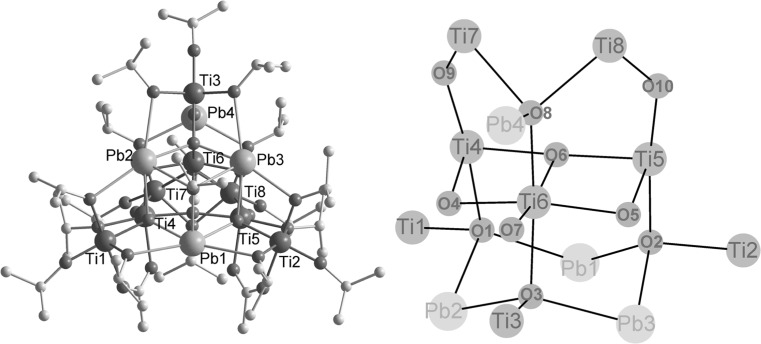
*Left* Molecular structure of Pb_4_Ti_8_O_10_(O*i*Pr)_18_(OAc)_2_ (**Pb4Ti8**). Hydrogen atoms are omitted for clarity. *Right* Cluster core

In an (unsuccessful) attempt to synthesize **Pb2Ti2** from Pb(OAc)_2_ and Ti(O*i*Pr)_4_ according to the literature [5] colourless crystals of Pb_4_Ti_8_O_10_(O*i*Pr)_18_(OAc)_2_ (**Pb4Ti8**) (Fig. 3; Table 2) were obtained after one year. This is of course no viable synthesis method; the structure of **Pb4Ti8** is nevertheless included in this paper to demonstrate the structural richness of Pb/Ti oxo clusters and to discuss some construction principles. Its cluster core can again formally be split in three layers of metals connected through oxygen atoms. The cluster core is approximately mirror-symmetric, with the mirror plane through Pb(1), Ti(3), Ti(6), and Pb(4) and perpendicular to the three metal layers. The overall symmetry of the cluster is lower than that of the cluster core due to the orientation of the peripheral isopropyl groups.

**Table 2:** Selected bond lengths/Å and angles/° for **Pb4Ti8**

Metal–metal distances	
Pb(1)–Pb(2) 3.8287(4)	Ti(4)–Ti(5) 3.822(1)
Pb(1)–Pb(3) 3.8332(4)	Ti(4)–Ti(6) 2.911(1)
Pb(2)–Pb(3) 3.6733(11)	Ti(5)–Ti(6) 2.910(1)
Pb(4)–Ti(7) 3.3100(10)	Ti(7)–Ti(8) 3.035(1)
Pb(4)–Ti(8) 3.3268(10)	
Metal-*μ*_4_-O distances	
Pb(1)–O(1) 2.306(4)	Pb(3)–O(2) 2.396(4)
Pb(2)–O(1) 2.398(4)	Pb(1)–O(2) 2.299(4)
Ti(1)–O(1) 1.978(4)	Ti(2)–O(2) 1.986(4)
Ti(4)–O(1) 2.050(4)	Ti(5)–O(2) 2.053(4)
Pb(2)–O(3) 2.295(3)	Pb(4)–O(8) 2.237(3)
Pb(3)–O(3) 2.293(3)	Ti(6)–O(8) 1.955(4)
Ti(3)–O(3) 2.064(4)	Ti(7)–O(8) 2.118(4)
Ti(6)–O(3) 1.950(4)	Ti(8)–O(8) 2.112(3)
Metal-*μ*_3_-O distances	
Pb(2)–O(4) 2.388(3)	Pb(3)–O(5) 2.402(4)
Ti(4)–O(4) 1.862(4)	Ti(6)–O(5) 1.938(4)
Ti(6)–O(4) 1.949(3)	Ti(5)–O(5) 1.866(4)
Ti(5)–O(6) 1.917(4)	Pb(4)–O(7) 2.387(4)
Ti(6)–O(6) 2.027(4)	Ti(3)–O(7) 1.867(4)
Ti(4)–O(6) 1.921(4)	Ti(6)–O(7) 1.932(4)
Metal-*μ*_2_-O distances	
Ti(4)–O(9) 1.852(4)	Ti(5)–O(10) 1.844(4)
Ti(7)–O(9) 1.812(4)	Ti(8)–O(10) 1.814(4)
Metal-*μ*_3_-O*i*Pr distances	
Pb(1)–O(11) 2.295(4)	Pb(4)–O(12) 2.508(4)
Pb(2)–O(11) 2.622(4)	Ti(7)–O(12) 2.176(4)
Pb(3)–O(11) 2.620(4)	Ti(8)–O(12) 2.175(4)
Metal-*μ*_4_-O-metal angles	
M–O(1)–M 99.4(2)–126.1(2)	M–O(3)–M 95.3(2)–118.0(2)
M–O(2)–M 99.7(1)–125.1(2)	M–O(8)–M 91.7(1)–128.4(2)
Ti-*μ*_3_-O-Ti-metal angles	
Ti(4)–O(6)–Ti(5) 169.5(2)	Ti(5)–O(6)–Ti(6) 95.0(2)
Ti(4)–O(6)–Ti(6) 95.0(2)	
Metal-*μ*_3_O*i*Pr-metal angles	
Pb(1)–O(11)–Pb(2) 102.07(14)	Pb(4)–O(12)–Ti(7) 89.66(13)
Pb(1)–O(11)–Pb(3) 102.29(14)	Pb(4)–O(12)–Ti(8) 90.26(14)
Pb(2)–O(11)–Pb(3) 97.24(13)	Ti(7)–O(12)–Ti(8) 88.48(14)

**Fig. 4:**
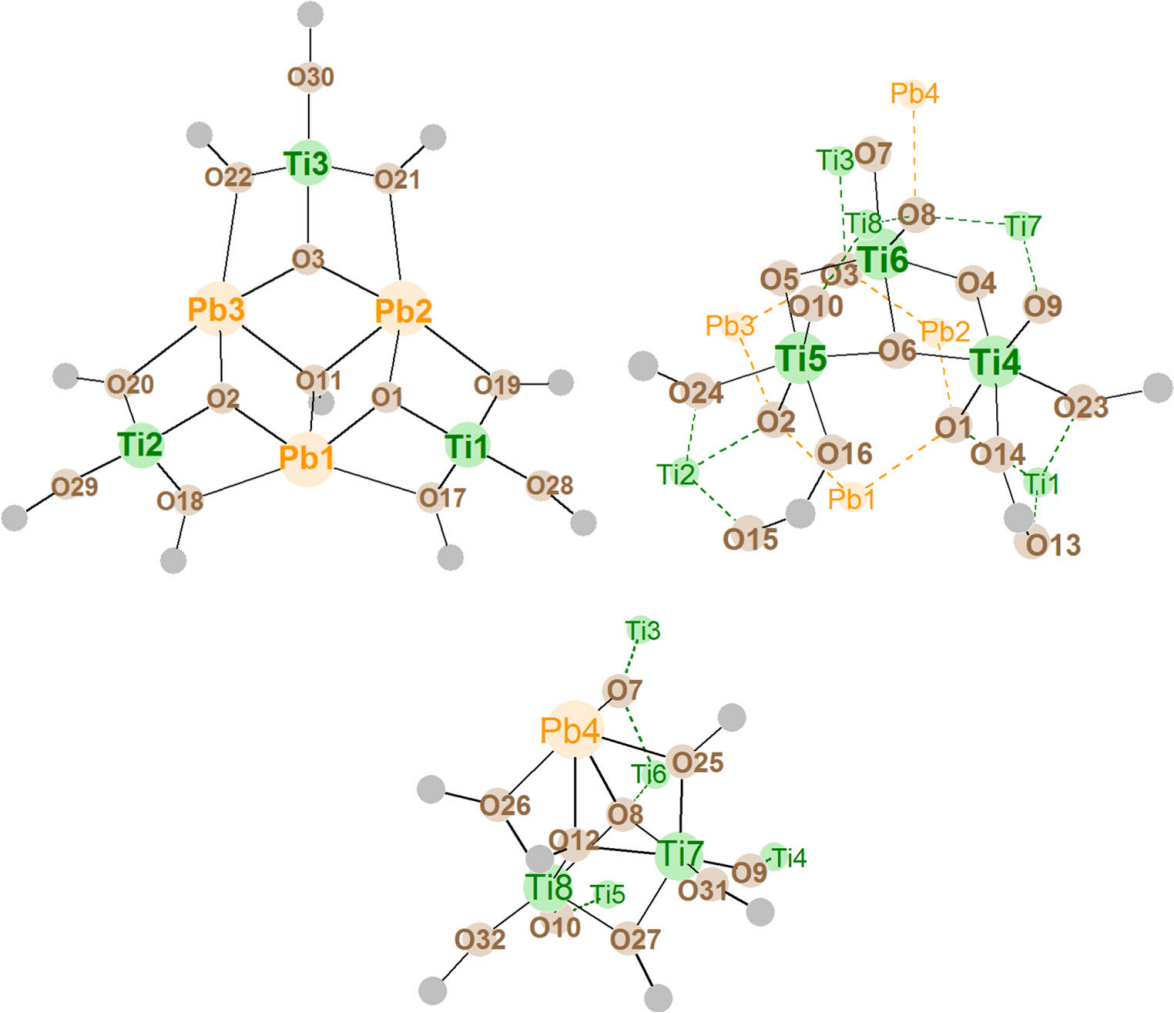
Schematic drawing of the layers in **Pb4Ti8**. *Dashed lines* indicate bonds to metals of adjacent layers

The bottom layer (Fig. 4) is formed by a Pb_3_O_3_ ring (Pb(1)–Pb(3) and O(1)–O(3)) which is capped by a rare *μ*_3_-O*i*Pr group (O(11)). The Pb^2+^ lone pairs are trans to the O(11) and thus point away from the Pb_3_O_3_ ring centre. The bond distance of the *μ*_3_-O*i*Pr group to Pb(1) (the Pb atom on the imagined mirror plane) is much shorter than to Pb(2) and Pb(3) [Pb(1)–O(11) 2.295(4) Å, Pb(2)–O(11) 2.622(4) Å, Pb(3)–O(11) 2.620(4) Å]. The ring oxygen atoms O(1) and O(2) are also shifted towards Pb(1) [Pb(1)–O(1) 230.6(4)/Pb(2)–O(1) 239.8(4) Å and Pb(1)–O(2) 229.9(4)/Pb(3)–O(2) 239.6(4) Å]. The asymmetry of the Pb_3_O(11) unit and the uneven distribution of the Pb–O bond lengths in the Pb_3_O_3_ ring is a consequence of the composition of the top layer, as will be discussed later. Three Ti atoms [Ti(1)–Ti(3)] are attached to the Pb_3_O_3_ ring through the ring oxygen atoms (Fig. 4). The Ti atoms are slightly above the plane of the Pb atoms, shifted towards the central layer. All Ti atoms of the bottom layer are additionally bonded to both neighbouring Pb atoms through *μ*_2_-O*i*Pr groups with relatively long Pb–O bond lengths [2.634(4)–2.870(4) Å]. Three terminal O*i*Pr groups (one on each Ti atom) complete the ligand sphere of Ti(1)–Ti(3).

The central layer (Fig. 4) consists of an open Ti_3_O triangle [Ti(4)–Ti(6) and O(6)] which is connected to the bottom layer through the three oxygen atoms of the Pb_3_O_3_ unit. These oxygen [O(1)–O(3)] atoms are thus *μ*_4_, connecting two Ti and two Pb atoms. Ti(4)–O(1) and Ti(5)–O(2) are about 0.1 Å longer than Ti(6)–O(3) [Ti(4)–O(1) 2.050(4) Å, Ti(5)–O(2) 2.053(4) Å, Ti(6)–O(3) 1.950(4) Å] thus compensating the shorter Pb(1)–O(1) and Pb(1)–O(2) bond distances in the bottom layer. The central oxygen atom O(6) of the Ti_3_O unit is nearly planar [sum of bond angles 356.5(5)], but its coordination is best described as T-shaped [Ti(4)–O(6)–Ti(5) 169.5(2)°, Ti(4)–O(6)–Ti(6) and Ti(5)–O(6)–Ti(6) 95.0(2)°]. Concomitantly, Ti(4)–O(6) and Ti(5)–O(6) are about 0.1 Å shorter than Ti(6)–O(6) [Ti(4)–O(6) 1.921(4) Å, Ti(5)–O(6) 1.917(4) Å, Ti(6)–O(6) 2.027(4) Å]. This is not only a consequence of the asymmetry of the bottom layer, but also of the unsymmetrical substitution of the Ti_3_O unit. A *μ*_3_-O connects Ti(4), Ti(6), and Pb(2), as well as Ti(5), Ti(6) and Pb(3) [O(4) and O(5), respectively]. There is no equivalent group connecting Ti(4) and Ti(5). Instead, Ti(4) and Ti(5) are bonded to the bottom layer [to Ti(1) and Ti(2)] through the two acetate bridges and two bridging O*i*Pr groups. Both have shorter Ti–O bond lengths to the Ti atoms of the central layer, Ti(4) and Ti(5). While Ti(4) and Ti(5) are octahedrally coordinated, Ti(3) has only a coordination number of 5 with a trigonal bipyramidal coordination geometry. This is quite unusual for Ti, as it prefers an octahedral coordination. The central Ti(6) of the Ti_3_O unit is coordinated to *μ*_3_-O atom which connects the central layer to Ti(3) of the bottom layer and Pb(4) of the top layer. There are no bridging O*i*Pr or carboxylate ligands within the central layer, and between the central and the top layer.

The top layer (Fig. 4) contains two Ti and one Pb atom, which are bridged by a *μ*_3_-O*i*Pr group [O(12)]. This O*i*Pr group is perpendicular to the Ti_2_Pb plane and mirrors the *μ*_3_-O*i*Pr group of the bottom layer. Pb(4), Ti(7), and Ti(8) are additionally connected through *μ*_4_-O(8), which also binds to Ti(6). The Ti–O bond lengths of Ti(7) and Ti(8) to O(8) are relatively long [Ti(7)–O(8) 2.118(4) Å, Ti(8)–O(8) 2.112(3) Å], whereas Ti(6)–O(8) is much shorter [1.955(4) Å]. In addition to O(7), which links all three layers, two *μ*_2_-oxygen atoms bridge Ti atoms of the top layer and the central layer [Ti(4)–O(9)–Ti(7) and Ti(5)–O(10)–Ti(8)].

Every metal of the top layer is connected to both neighbouring metals through a *μ*_2_-O*i*Pr group. The Ti–O bonds of Pb–Ti bridging alkoxo groups are about 0.1 Å shorter than those bridging Ti–Ti [Ti(7)–O(25) 1.946(4) Å, Ti(8)–O(26) 1.944(4) Å, Ti(7)–O(27) 2.045(4) Å, Ti(8)–O(27) 2.037(4) Å]. Two terminal O*i*Pr groups complete the octahedral coordination sphere of Ti(7) and Ti(8).

## Conclusions

The structures of the previously reported Pb_2_Ti_x_ (*x* = 2, 4, 6, 8) oxo clusters are based on structural motifs typical of monometallic titanium oxo clusters (see “Introduction”). This is no longer the case when the number of Pb^2+^ ions is increased. The high tendency of the [TiO_6_] octahedra to connect with each other is also observed in **Pb6Ti6** and **Pb4Ti8**. The same is true for the Pb/O polyhedra (with different coordination numbers), where the Pb^2+^ lone pair is stereochemically active. The preferred condensation of polyhedra of the same kind is probably due to the different ionic radii of Ti(IV) and Pb(II) and especially pronounced in **Pb6Ti6**. Six [TiO_6_] octahedra in **Pb6Ti6** form a planar six-membered ring, which is capped by Pb_3_ units from above and below. In **Pb4Ti8**, five [TiO_6_] octahedra connect with each other in a three-dimensional arrangement (the center and top layer) to which one isolated Pb/O polyhedron is condensed. This PbTi_5_ unit is attached to a Pb_3_O_3_ ring (the bottom layer), to which two isolated [TiO_6_] octahedra and one [TiO_5_] trigonal bipyramid [Ti(3)] are bonded.

## Experimental

All experiments were carried out under Ar atmosphere using standard Schlenk techniques. Pb(OAc)_2_·2 H_2_O was obtained from Merck and Ti(O*i*Pr)_4_ from ABCR. Water-free lead acetate was obtained by drying in vacuum at 130 °C over night. The drying process was monitored by IR spectroscopy.

### PbTi oxo clusters

#### Pb_6_Ti_6_O_9_(OAc)(OMc)_17_ (Pb6Ti6)

Pb(OAc)_2_ (651 mg, 2 mmol), 568 mg of Ti(O*i*Pr)_4_ (2 mmol), and 278 mg of dry allylic alcohol (4 mmol) were heated for 2 h at 70 °C. The clear solution was allowed to cool to room temperature and then 1.62 g of methacrylic acid (18.9 mmol) was added. A white precipitate was formed, which disappeared after 1 h. Colourless crystals of Pb_6_Ti_6_O_9_(OAc)(OMc)_17_ were obtained after four months. Yield: 39 mg (34% rel. Ti).

#### Pb_4_Ti_8_O_10_(OiPr)_18_(OAc)_2_ (Pb4Ti8)

It was attempted to synthesize Pb_2_Ti_2_O(O*i*Pr)_8_(OAc)_2_ according to the literature [5]. Dry Pb(OAc)_2_ (1.306 g, 4 mmol) and 3.41 g of Ti(O*i*Pr)_4_ (12 mmol) were stirred in 20 cm^3^ of dry *n*-hexane. After 3 days [because of the low solubility of Pb(OAc)_2_] a clear solution was obtained. After two weeks the solution was concentrated to 15 cm^3^. Colourless crystals were obtained after 1 year, beside much white precipitate.

### X-ray crystallography

Crystallographic data were collected on a Bruker AXS SMART APEX II four-circle diffractometer with κ-geometry at 100 K using MoK_α_ (*λ* = 0.71073 Å) radiation. The data were corrected for polarization and Lorentz effects, and an empirical absorption correction (SADABS) was employed. The cell dimensions were refined with all unique reflections. SAINT PLUS software (Bruker Analytical X-ray Instruments, 2007) was used to integrate the frames. Symmetry was checked with the program PLATON.

The structures were solved by charge flipping (JANA2006). Refinement was performed by the full-matrix least-squares method based on *F* ^2^ (SHELXL97 [10]) with anisotropic thermal parameters for all non-hydrogen atoms. Hydrogen atoms were inserted in calculated positions and refined riding with the corresponding atom. Crystal data, data collection parameters and refinement details are listed in Table 3.

**Table 3:** Crystal data, data collection parameters and refinement details

	**Pb6Ti6**	**Pb4Ti8**
Empirical formula	C_73_H_93_O_46_Pb_6_Ti_6_	C_58_H_132_O_32_Pb_4_Ti_8_
*M*_r_	3237.0	2553.6
Crystal system	Monoclinic	Monoclinic
Space group	*P*2_1_ */n*	*P*2_1_ */n*
*a*/Å	14.170(2)	14.459(1)
*b*/Å	19.755(3)	26.347(2)
*c*/Å	35.927(5)	23.735(2)
*ß*/°	90.010(2)	90.086(4)
*V*/Å^3^	10,057(2)	9042(1)
*Z*	4	4
*D*_*x*_/Mg m^−3^	2.138	1.876
*Μ*/mm^−1^	10.538	8.152
Crystal size/mm	0.32 × 0.24 × 0.12	0.41 × 0.32 × 0.28
No. of ind., obs. refl.	31,237, 31,237	24,486, 19,725
Criterion for obs. refl.	*I* > 2*σ*(*I*)	*I* > 2*σ*(*I*)
*Θ*_min, max_/°	1.86, 30.77	1.15, 29.28
*R*[*F* ^2^ > 2*σ* (*F*)]*, R*_w_(*F* ^2^)*, S*	0.027, 0.033, 1.197	0.039, 0.060, 1.072
Weighting scheme*	*x* = 0.0064*, y* = 37.333	*x* = 0*, y* = 113.913
Δ*ρ*_max_/e Å^−3^	1.51, −1.54	3.55, −2.29

* *ω* = 1*/σ* ^2^(*F*_0_^2^) + (*xP*)^2^ + *yP*], where *P* = (*F*_0_^2^ + 2 *F*_c_^2^)*/*3

CCDC 1530015 (**Pb6Ti6**) and 1530016 (**Pb4Ti8**) contain supplementary crystallographic data. The data can be obtained free of charge from The Cambridge Crystallographic Data Centre via http://www.ccdc.cam.ac.uk/data_request/cif.
